# Pre-treatment T-cell subsets associate with fingolimod treatment responsiveness in multiple sclerosis

**DOI:** 10.1038/s41598-019-57114-2

**Published:** 2020-01-15

**Authors:** Mahtab Ghadiri, Ayman Rezk, Rui Li, Ashley Evans, Paul S. Giacomini, Michael H. Barnett, Jack Antel, Amit Bar-Or

**Affiliations:** 1 Montreal Neurological Institute, McGill University, Montreal, QC Canada; 2 Brain and Mind Centre, University of Sydney, Camperdown, NSW Australia; 3 Center for Neuroinflammation and Experimental Therapeutics, and Department of Neurology, Perelman School of Medicine, University of Pennsylvania, Philadelphia, PA USA; 4 Taylor Fry, Sydney, NSW Australia

**Keywords:** Autoimmune diseases, Immunosuppression, Lymphocytes, Predictive markers, Multiple sclerosis

## Abstract

Biomarkers predicting fingolimod (FTY) treatment response in relapsing-remitting multiple sclerosis (RRMS) are lacking. Here, we performed extensive functional immunophenotyping using multiparametric flow cytometry to examine peripheral immune changes under FTY treatment and explore biomarkers of FTY treatment response. From among 135 RRMS patients who initiated FTY in a 2-year multicentre observational study, 36 were classified as ‘Active’ or ‘Stable’ based on clinical and/or radiological activity on-treatment. Flow cytometric analysis of immune cell subsets was performed on pre- and on-treatment peripheral blood mononuclear cells (PBMC) samples. Decreased absolute counts of B cells and most T-cell subsets were seen on-treatment. Senescent CD8 + T cells, CD56 + T cells, CD56^dim^ natural killer cells, monocytes and dendritic cells were not reduced in number and hence relatively increased in frequency on-treatment. An unbiased multiparametric and traditional manual analysis of T-cell subsets suggested a higher pre-treatment frequency of CD4 + central memory T cells (TCM) in patients who were subsequently Active versus Stable on-treatment. Lower pre-treatment terminally differentiated effector memory (TEMRA) cell frequencies were also seen in the subsequently Active cohort. Together, our data highlight differential effects of FTY on peripheral immune cell subsets and suggest that pre-treatment T-cell subset frequencies may have value in predicting FTY treatment response.

## Introduction

Fingolimod (FTY) was shown to reduce relapse rates in relapsing-remitting multiple sclerosis (RRMS) by approximately 50% and diminish development of new or enlarging brain lesions on magnetic resonance imaging (MRI) by 74%^1^. It is believed to act principally through interference with immune cell trafficking. Immune cells expressing the lymph node (LN) homing receptor CCR7 require sphingosine-1-phosphate (S1P) binding to exit LNs. FTY causes internalization of the S1P1 receptor resulting in immune cell sequestration within LNs^2^. This sequestration is believed to reduce the migration of pathogenic T cells into the central nervous system (CNS) of MS patients^2,3^, although the degree of FTY treatment response has not been found to correlate with the degree of overall lymphopenia (total lymphocyte counts; TLC) induced by treatment. Distinct T-cell subsets are differentially impacted by FTY. A preferential reduction in circulating CCR7-expressing subsets has been firmly established^3,4^, however effects on other putatively MS-relevant T-cell subsets have been incompletely elucidated.

S1P receptor antagonism affects migration patterns of other MS-implicated immune cell subsets, including B cells, natural killer (NK) cells and dendritic cells (DC)^3,5^. Additionally, S1P signalling may affect the differentiation of MS-relevant T-cell subsets, including T-helper (Th)-17 and regulatory T cells (Tregs)^6^.

Rare but serious drug-related adverse events have emerged over time with the use of FTY in MS patients, including cases of cryptococcal meningitis, progressive multifocal leukoencephalopathy and immune thrombocytopenic purpura^7–9^. Biomarkers predicting such adverse events or distinguishing patients who respond particularly well or poorly to FTY would be of great clinical value but are yet to be discovered. For other MS disease-modifying therapies (DMTs), variable treatment responses have been linked to differences in pre-treatment immune abnormalities. For example, heterogeneity in baseline type I interferon (IFN) and Th17 activity may explain variability in IFN-β responsiveness^10,11^.

From a prospectively followed cohort of FTY-treated patients, we selected patients who either exhibited new disease activity while on-treatment (deemed ‘Active’) or did not (‘Stable’). We examined FTY treatment effects on a range of potentially MS-relevant immune cell subsets in all patients, and then assessed whether pre-treatment phenotypic or functional measures of immune cell subsets were associated with the development of disease activity while on FTY.

## Results

### Demographic and clinical characteristics

The characteristics of the 36 participants are outlined in Table 1. The Active and Stable cohorts were well-matched for age, MS duration, pre-treatment Expanded Disability Status Score (EDSS) and pre-treatment 2-year annualized relapse rate (ARR). There were more females in the Stable compared with the Active cohort (94% vs. 67%). As FTY is approved as a second-line agent in Canada, most subjects had previously received a DMT. The on-treatment disease activity measures (relapses and new or enlarging T2 hyperintense lesions; NEL) distinguishing the cohorts are outlined in Table 1. The mean number of NEL identified on follow-up MRI in the Active cohort was 12 (range 1–31) and the majority (56%) also experienced a clinical relapse on-treatment.

**Table 1:** Patient characteristics.

	All patients (n = 36)	Active cohort (n = 18)	Stable cohort (n = 18)
**Baseline characteristics**			
Age (mean, range)	37 (22–54)	36.1 (22–54)	38.1 (24–49)
Female (%, number)	81% (29/36)	67% (12/18)	94% (17/18)
Duration of MS (mean, range)	10.8 (0–29)	11.5 (1–29)	10.2 (0–21)
Previously treated (%, number)	97% (35/36)	100% (18/18)	94% (17/18)
ARR (mean)	1.0	1.0	1.1
EDSS (mean, range)	2.7 (0–6.0)	2.9 (1.0–5.5)	2.4 (0–6.0)
**Disease activity on FTY**			
Clinical relapse/s (%, number)	28% (10/36)	56% (10/18)	None
NEL (mean, range)	5.5 (0–31)	12 (1–31)	0 (0–1)
≥5 NEL (%, number)	31% (11/18)	61% (11/18)	None

Baseline demographics and multiple sclerosis (MS) disease details (duration, previous treatment, 2-year annualized relapse rate (ARR) and expanded disability status score (EDSS)) are shown in the first part of the table. The proportion of patients developing one or more clinical relapses, the mean number of new or enlarging T2 MRI lesions (NEL), and the proportion of patients developing five or more NEL over the 24-month study period are shown in the second part of the table. Data are shown for all patients combined and for Active and Stable cohorts separately.

### FTY treatment effects on major immune cell subsets

Analysis of absolute counts of major immune cell subsets pre- and on-treatment (Table 2) revealed the expected reduction in TLC and total CD3 + T-cell, CD4 + T-cell, CD8 + T-cell and CD20 + B-cell counts. CD3 + cells co-expressing CD56 were not reduced in number on-treatment. While CD56^bright^ NK cells decreased, the number of CD56^dim^ NK cells was not affected by treatment. There were no decreases in absolute counts of monocytes or DC subsets.

**Table 2:** Changes in major immune cell subset absolute counts On-treatment versus Pre-treatment with FTY.

Cell subset	Pre mean (SD)	On mean (SD)	Change (On – Pre)	*p* value (unadjusted)	*p* value (adjusted)
TLC	2043 (894)	435 (196)	−1608	<0.0001	<0.0059
Total CD3+	1650 (695)	250 (210)	−1400	<0.0001	<0.0059
CD4+	1124 (487)	66 (84)	−1058	<0.0001	<0.0059
CD8+	435 (255)	129 (121)	−306	<0.0001	<0.0059
Total B cells	285 (235)	19 (20)	−266	<0.0001	<0.0059
Total NK cells	135 (113)	118 (126)	−17	ns	ns
CD56^bright^ NK	19 (19)	3 (2)	−16	0.0007	0.0413
CD56^dim^ NK	89 (82)	95 (114)	6	ns	ns
Monocytes	551 (179)	501 (139)	−50	ns	ns
TNFα + monocytes	167 (48)	120 (81)	−47	ns	ns
Total DC	200 (204)	195 (121)	−4	ns	ns
HLA-DR + CD11c-CD123 + DC	16 (9)	12 (7)	−4	0.0366	ns
HLA-DR^hi^ CD11c + DC	22 (17)	20 (14)	−2	ns	ns
HLA-DR^dim^ CD11c + DC	110 (83)	156 (106)	46	ns	ns
CD3 + CD56+	72 (89)	79 (136)	7	ns	ns
CD4 + CD56 +	1 (3)	10 (34)	9	ns	ns

Mean and standard deviation (SD) of absolute cell counts pre-treatment (Pre) and on-treatment (On) for major immune cell subsets are shown in cells/µL (n = 10–33). The differences between on-treatment and pre-treatment counts are shown. The results of paired t-tests comparing the two timepoints are shown both unadjusted and after Bonferroni correction for multiple comparisons. ns = not significant.

**Figure 1:**
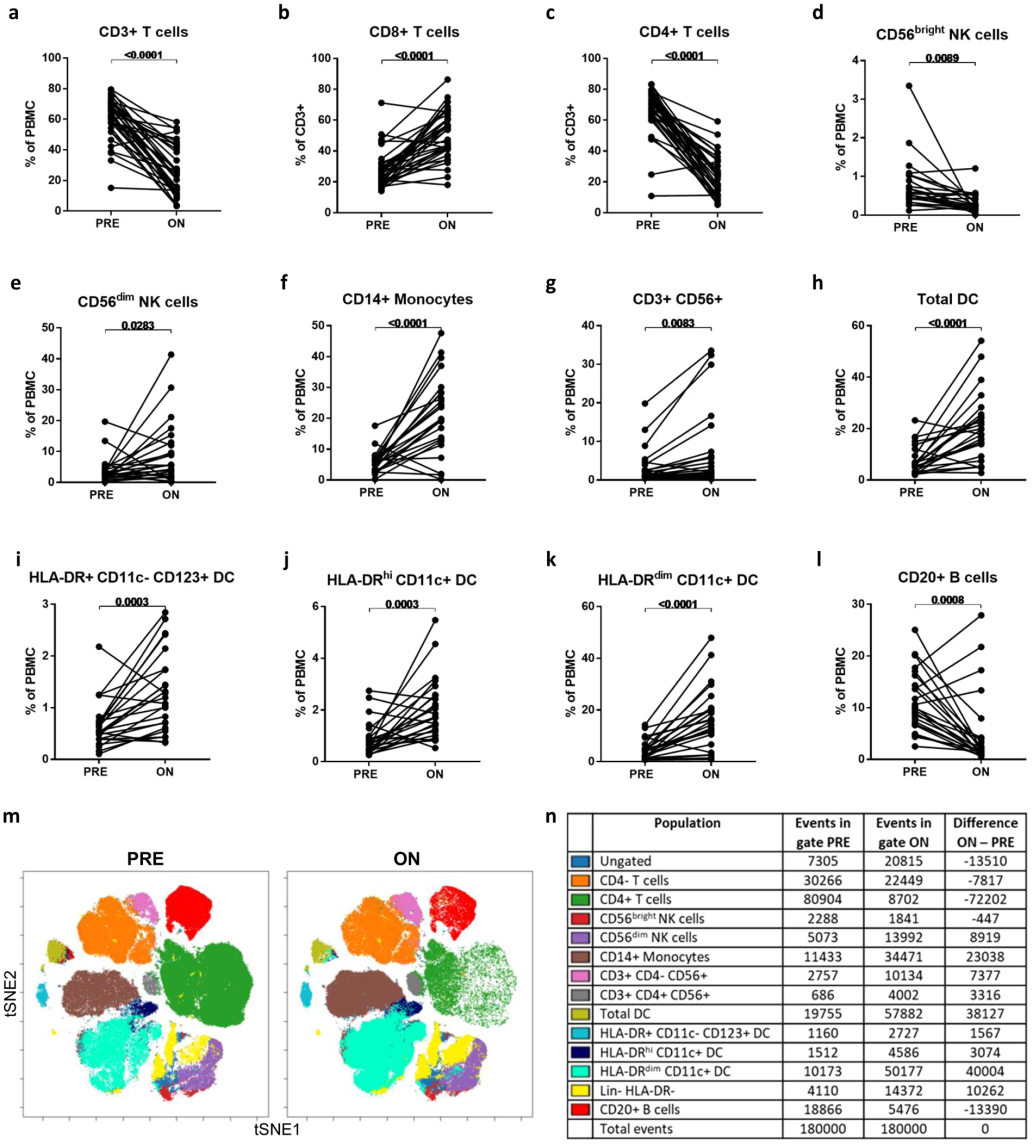
Major immune cell subset changes On-treatment with FTY compared to Pre-treatment. Frequencies of major immune subsets were compared in pre- and on-treatment samples using paired *t*-tests (**A–L**). Combined down-sampled data from six patients’ matched pre- and on-treatment samples is shown using a viSNE analysis and displayed with overlaid manually drawn population gates (**M**). Event counts within each gate on the viSNE plot are shown for pre- and on-treatment timepoints and the difference between the two timepoints is indicated (N). Lin = lineage markers (CD3, CD14, CD56, CD16, CD20).

The resultant shifts in major immune cell subset frequencies within peripheral blood mononuclear cells (PBMC) are shown in Fig. 1. These frequency data are in keeping with the analysis of absolute counts in that lymphocyte populations which reduced significantly in counts also reduced in frequency, while immune cell populations which remained relatively stable in absolute counts were increased in relative frequency within total PBMC. Specifically, the frequency of CD3 + T cells within PBMC was reduced on FTY treatment (61% pre-treatment to 26% on-treatment, *p* < 0.0001; Fig. 1A). Amongst CD3 + T cells, the frequency of CD8 + T cells increased (27% pre- to 51% on-treatment, *p* < 0.0001; Fig. 1B) as frequencies of CD4 + T cells decreased (66% pre- to 23% on-treatment, *p* < 0.0001; Fig. 1C). The small CD56^bright^ subset of NK cells decreased in frequency within PBMC (0.8% pre- to 0.3% on-treatment, *p* = 0.0089; Fig. 1D), while the more abundant CD56^dim^ NK cells increased in frequency (3.8% pre- to 9.1% on-treatment, *p* = 0.0283; Fig. 1E) with treatment. The relative frequencies of monocytes increased on FTY (6% pre- to 21% on-treatment, *p* < 0.0001; Fig. 1F), as did the frequency of CD3 + cells expressing CD56 + (3.3% pre to 7.3% on-treatment, *p* = 0.0083; Fig. 1G). Total DC, as well as the DC subsets defined as HLA-DR + CD11c-CD123+, HLA-DR^hi^CD11c+ and HLA-DR^dim^CD11c+, increased as a proportion of PBMC on FTY treatment (Fig. 1H–K). The frequency of B cells within total PBMC decreased with treatment (11% pre- to 5% on-treatment, *p* = 0.0008; Fig. 1L). The results of a viSNE analysis using combined down-sampled data from six patients’ matched pre- and on-treatment samples is displayed with overlaid manually drawn population gates (Fig. 1M). Event counts within each gate on the viSNE plot (Fig. 1N) show relatively fewer T cells, B cells and CD56^bright^ NK cells and relative increases in the other immune cell subset counts on-treatment, in line with the results from the traditional analyses.

### Effector memory and senescent T cells predominate in the circulation of FTY-treated patients

**Figure 2:**
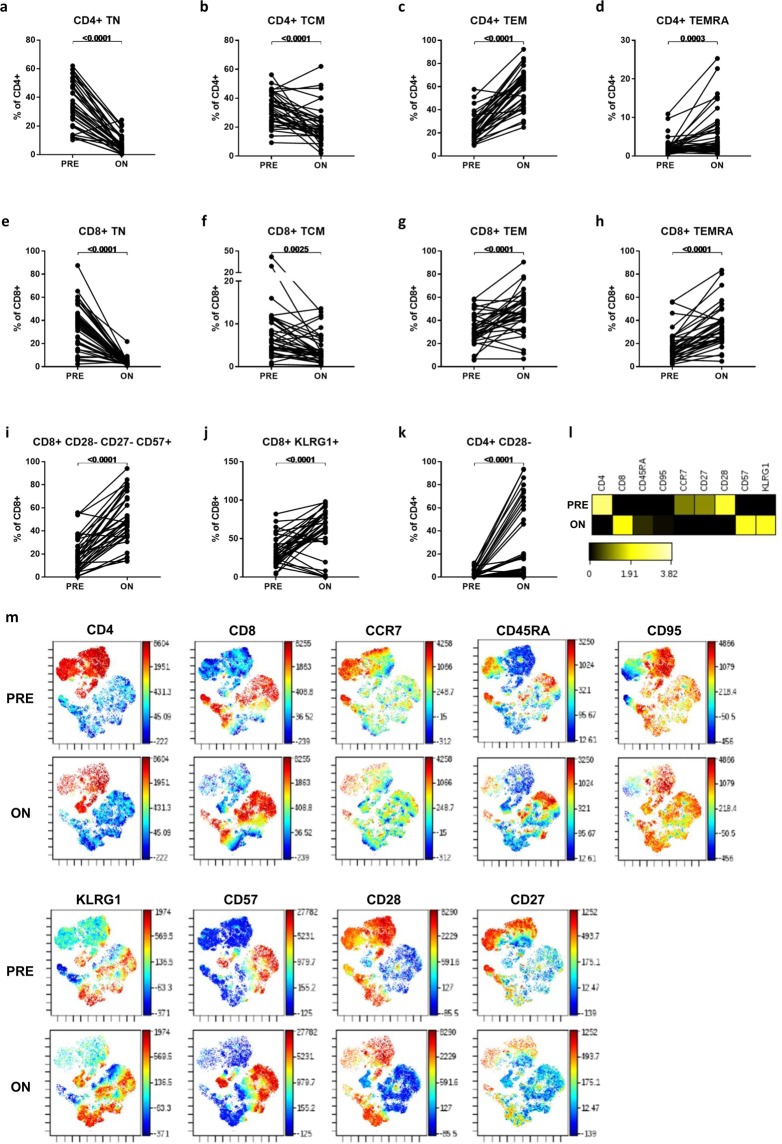
Naïve, memory and senescent T-cell subset changes On-treatment with FTY compared to Pre-treatment. Frequencies of naïve, memory and senescent T-cell subsets within total CD4 + and CD8 + T cells were compared in pre- and on-treatment samples using paired *t*-tests (**A–K**). Combined down-sampled data from nine patients’ matched pre- and on-treatment samples was analysed using viSNE, gating on CD3 + T cells. Relative expression of each marker within the total CD3 + population is shown in the heatmap (**L**), which displays the fold-change of each marker relative to the lesser of the two time points. viSNE plots (tSNE1 and tSNE2 plotted on x and y axes respectively) coloured by each marker (**M**) show the treatment-induced changes in the expression of these molecules across the CD4 + and CD8 + clusters.

Naïve and memory T-cell subsets all reduced in absolute numbers on-treatment (Table 3), although reductions in the least abundant CD8+ subsets (central memory; TCM and terminally differentiated effector memory; TEMRA) were insignificant with Bonferroni correction. As expected, larger reductions in absolute counts were seen among CCR7+ populations (TCM and naïve; TN), which all decreased by >90% of pre-treatment counts, compared with CCR7- effector memory (TEM) and TEMRA subsets, which decreased by between 32–83% of pre-treatment counts. Therefore, among both total CD4 + T cells (Fig. 2A–D) and total CD8+ T cells (Fig. 2E–F), the CCR7+ sub-populations reduced in frequency, while the CCR7- sub-populations were reciprocally increased in frequency, on-treatment.

**Table 3:** Changes in major T-cell subset absolute counts On-treatment versus Pre-treatment with FTY.

	Cell subset	Pre mean (SD)	On mean (SD)	Change (On – Pre)	*p* value (unadjusted)	*p* value (adjusted)
Naïve and memory T cells	CD4+ TN	422 (268)	4 (8)	−418	<0.0001	<0.0059
	CD4+ TCM	357 (185)	11 (15)	−346	<0.0001	<0.0059
	CD4+ TEM	257 (165)	43 (71)	−214	<0.0001	<0.0059
	CD4+ TEMRA	22 (20)	4 (5)	−18	<0.0001	<0.0059
	CD8+ TN	158 (122)	4 (4)	−155	<0.0001	<0.0059
	CD8+ TCM	41 (62)	4 (4)	−37	0.0021	ns
	CD8+ TEM	139 (85)	61 (93)	−78	0.0001	0.0059
	CD8+ TEMRA	73 (80)	49 (61)	−23	0.0031	ns
Cytokine-expressing T cells	CD4+ IFNγ+	135 (84)	21 (41)	−114	<0.0001	<0.0059
	CD4+ GM-CSF+	123 (72)	8 (10)	−115	<0.0001	<0.0059
	CD4+ IL-17+	6 (4)	0 (0)	−6	<0.0001	<0.0059
	CD4+ IL-22+ *	11 (8)	1 (1)	−11	<0.0001	<0.0059
	CD4+ IL-4+ *	30 (19)	2 (2)	−28	<0.0001	<0.0059
	CD4+ IL-10+	9 (8)	1 (1)	−8	<0.0001	<0.0059
	CD8+ IFNγ+	147 (93)	89 (103)	−58	0.0006	0.0354
	CD8+ GM-CSF+	37 (34)	10 (10)	−27	<0.0001	<0.0059
	CD8+ IL-17+	2 (1)	2 (3)	0	ns	ns
	CD8+ IL-4+	12 (14)	3 (3)	−9	0.0003	0.0177
RTE & Senescent T cells	CD4+ RTE	393 (246)	6 (8)	−387	<0.0001	<0.0059
	CD8+ CD28- CD27- CD57+	80 (86)	71 (78)	−9	ns	ns
	CD8+ KLRG1+	131 (112)	79 (105)	−52	0.0126	ns
	CD4+ CD28−	18 (33)	32 (74)	14	ns	ns

Mean and standard deviation (SD) of absolute cell counts pre-treatment (Pre) and on-treatment (On) for major T-cell subsets are shown in cells/µL (n = 20–32). The differences between on- and pre-treatment counts are shown. The results of paired t-tests comparing the two timepoints are shown both unadjusted and after Bonferroni correction for multiple comparisons. ns = not significant.

We examined T cells at different stages of development. FTY significantly reduced the number (Table 3) of recent thymic emigrants (RTE; CD31 + CD45RO-) and their frequency (35% pre- to 13% on-treatment, *p* < 0.0001). Senescent CD8+ T cells (CD28-CD27-CD57+) were not decreased in number (Table 3) and therefore significantly increased in frequency within remaining CD8+ T cells on-treatment (19% pre- to 51% on-treatment, *p* < 0.0001; Fig. 2I). Similarly, both CD28-CD4 + T cells and CD8+ T cells expressing the senescence marker KLRG1 increased in frequency on-treatment (Fig. 2J,K).

A viSNE analysis including markers of naïve, memory and senescent T-cell subsets was also performed using combined down-sampled data from nine patients’ matched pre- and on-treatment samples. Relative expression of each marker within the total CD3 + population is shown in the heatmap (Fig. 2L), which displays the fold-change of each marker relative to the lesser of the two time points, and highlights the reduction in mean CD4, CCR7, CD27 and CD28 expression and the increase in mean CD8, CD57 and KLRG1 expression on-treatment. viSNE plots coloured by each marker (Fig. 2M) show the treatment-induced changes in the expression of these molecules across the CD4 + and CD8 + clusters.

**Figure 3:**
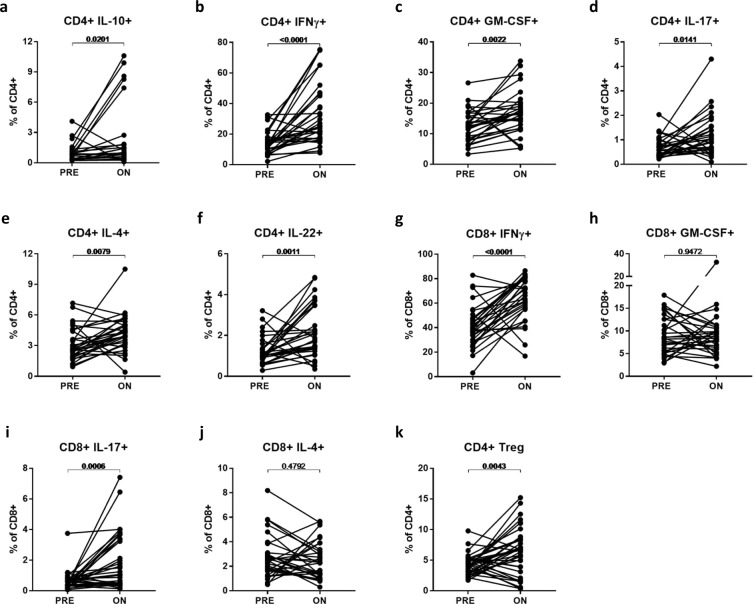
Changes in cytokine-expressing T-cell subsets and Tregs On-treatment with FTY compared to Pre-treatment. Frequencies of cytokine-expressing CD4+ (**A–F**) and CD8+ (**G–J**) T cells, as well as frequencies of CD4+ Tregs (**K**) were compared in pre- and on-treatment samples using paired *t*-tests.

A range of cytokine-expressing T-cell subsets were examined (Table 3); all were significantly reduced in absolute counts on-treatment, except for IL-17-expressing CD8 + T cells for which the number of events was very low. Despite these reductions, all CD4 + cytokine-expressing subsets and CD8 + T cell subsets expressing IFNγ and IL-17, increased in frequency within the respective total CD4 + and CD8 + T cells on-treatment (Fig. 3A–J). This increase in frequency of cytokine-expressing T cell subsets on-treatment may be accounted for by the relatively greater loss of naive (TN; mean 99% decrease in counts) which tend to express little cytokine, compared with memory subsets (mean 73% decrease in counts) on-treatment. Indeed within TEM (the predominant circulating subset on-treatment), no cytokine-expressing subsets increased in frequency but rather a decreased frequency of subsets expressing GM-CSF, IL-17 and IL-4 was seen (data not shown).

Additional potentially MS-relevant T-cell subsets examined are outlined in Table 4 (absolute counts) and Supplementary Fig. S1 (frequencies). Most T-cell subsets reduced in number on-treatment, including activated (HLA-DR + CD38+) and proliferating (Ki-67+) subsets, although these both increased in frequency amongst circulating CD4 + (but not CD8+) T cells on-treatment. CD8+ T cells with exhausted phenotypes (including Tbet^dim^EOMES^hi^ and cells expressing multiple inhibitory receptors) decreased to a lesser extent and hence increased as a proportion of residual circulating CD8+ T cells (CD8 + Tbet^dim^EOMES^hi^: 20% pre- to 37% on-treatment, *p* = 0.0027; CD8 + 2B4 + PD-1+: 8% pre- to 13% on-treatment, *p* = 0.0062; CD8 + 2B4 + PD-1 + CTLA-4+: 1.2% pre- to 1.6% on-treatment, *p* value not significant).

**Table 4:** Changes in other T-cell subset absolute counts On-treatment versus Pre-treatment with FTY.

	Cell subset	Pre mean (SD)	On mean (SD)	Change (On – Pre)	*p* value (unadjusted)	*p* value (adjusted)
Regulatory T cells	Total Treg	45 (23)	3 (3)	−43	<0.0001	<0.0059
	FoxP3+ Treg	38 (20)	2 (3)	−36	<0.0001	<0.0059
	RTE Treg	10 (8)	0 (0)	−10	<0.0001	<0.0059
	Memory Treg	27 (15)	2 (2)	−25	<0.0001	<0.0059
	CD39+ Treg	16 (11)	2 (2)	−14	<0.0001	<0.0059
	Helios + Treg	34 (17)	2 (3)	−32	<0.0001	<0.0059
	Tigit + Treg	19 (10)	1 (1)	−18	<0.0001	<0.0059
	HLA-DR + Treg	6 (4)	1 (1)	−6	<0.0001	<0.0059
	PD-1+ Treg	3 (2)	0 (0)	−2	<0.0001	<0.0059
Activated & proliferating T cells	CD4+ HLA-DR + CD38+	7 (4)	1 (1)	−6	<0.0001	<0.0059
	CD4+ Ki67+	14 (11)	2 (2)	−12	<0.0001	<0.0059
	CD8+ HLA-DR+ CD38+	10 (12)	3 (3)	−8	<0.0001	<0.0059
	CD8+ Ki67+	8 (5)	2(2)	−5	<0.0001	<0.0059
Exhausted T-cells	CD8+ Tbet^dim^ EOMES^hi^	102 (76)	77 (112)	−25	ns	ns
	CD8+ PD-1+ 2B4+	64 (60)	22 (17)	−42	0.0033	ns
	CD8+ PD-1+ 2B4+ CTLA-4+	7 (6)	2 (2)	−5	0.0002	0.0118
Other T cells of interest	CD4+ MCAM+	17 (16)	1 (2)	−16	<0.0001	<0.0059
	CD8+ MCAM+	8 (5)	3 (6)	−5	<0.0001	<0.0059
	CD4+ CD62L+	406 (286)	19 (41)	−499	<0.0001	<0.0059
	CD4+ CD95+	495 (272)	51 (69)	−444	<0.0001	<0.0059
	CD8+ CD95+	228 (166)	108 (107)	−120	<0.0001	<0.0059

Mean and standard deviation (SD) of absolute cell counts pre-treatment (Pre) and on-treatment (On) for T-cell subsets are shown in cells/µL (*n* = 17–32). The differences between on-treatment and pre-treatment counts are shown. The results of paired *t*-tests comparing the two timepoints are shown both unadjusted and after Bonferroni correction for multiple comparisons. ns = not significant.

### FTY alters the regulatory versus effector T-cell balance

We explored the effect of FTY on CD4 + Tregs (defined as CD4^+^CD25^hi^CD127^lo^CD3^+^ cells) and on Treg subsets expressing markers previously associated with suppressive capacity, including FoxP3, CD39, Helios, Tigit, HLA-DR and PD-1. All subsets showed a significant decrease in counts on-treatment (Table 4). Overall, the average frequency of Treg among circulating CD4 + T cells tended to increase on-treatment with FTY (6.3%) compared to pre-treatment (4.2%; *p* = 0.0043; Fig. 3K) consistent with a prior report by Haas *et al*.^12^, though we noted a substantial minority (39%) of patients in the present study appeared to have slight decreases or no obvious change in Treg frequencies on-treatment. Both RTE (CD31 + CD45RO-) Tregs and memory (CD45RO + ) Tregs decreased in absolute numbers (Table 4); these changes resulted in a significant increase in memory Treg frequency (2.7% pre- to 5.7% on-treatment, *p* < 0.0001), yet a significant decrease in RTE Treg frequency (0.8% pre- to 0.4% on-treatment, *p* < 0.0001; Supplementary Fig. S2) within total CD4 + T cells. Tregs expressing CD39, a marker associated with memory Tregs^13^, showed a corresponding increase in frequency on-treatment (1.5% pre- to 3.6% on-treatment, *p* = 0.0001; Supplementary Fig. S2). HLA-DR + Tregs, a subset of mature Tregs reported to mediate early cell contact-dependant suppression^14^, also increased in frequency on-treatment (0.7% pre- to 2.4% on-treatment, *p* < 0.0001; Supplementary Fig. S2). The frequency of Treg subsets expressing other markers associated with suppressive capacity (FoxP3, Tigit, Helios and PD-1) remained unchanged within total CD4+ T cells on-treatment (Supplementary Fig. S2).

**Figure 4:**
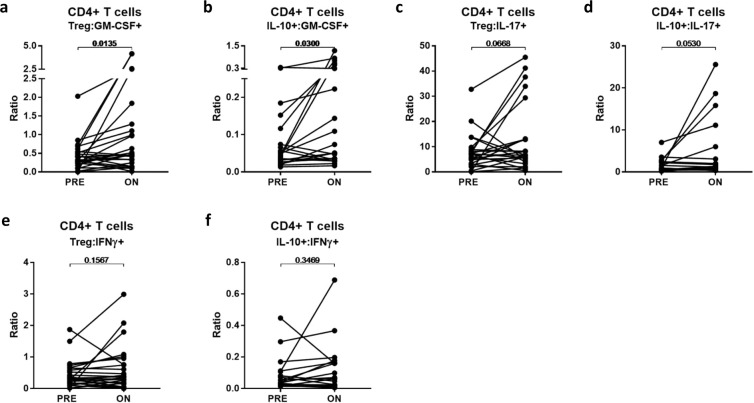
Changes in regulatory:effector T-cell ratios On-treatment with FTY compared to Pre-treatment. Ratios of presumed regulatory versus pro-inflammatory cytokine-expressing CD4+ T cells were compared in pre- and on-treatment samples using paired *t*-tests (**A–F**).

We assessed the impact of FTY treatment on ratios of regulators (Tregs and IL-10-expressing CD4+ T cells) to several pro-inflammatory effector CD4+ T-cell subsets. We observed either significant increases or trends towards increases in the ratios of regulators to CD4+ effector subsets expressing GM-CSF and IL-17, but not to those expressing IFNγ(Fig. 4A–F3L–Q).

### T-cell trafficking and adhesion molecule expression on FTY treatment

Given FTY’s effects on lymphocyte recirculation, we examined T-cell subsets expressing molecules implicated in CNS migration and entry. MCAM-expressing CD4+ and CD8+ T cells were reduced in number (Table 4), though CD4 + MCAM + cells increased in frequency on-treatment (1.7% pre- to 2.7% on-treatment, *p* = 0.0084; Supplementary Fig. S1). The level of expression of other adhesion molecules and integrins (CD11a/LFA-1, CD6, CD162/PSGL-1, CD49d/α4-integrin) was generally higher on circulating T cells on-treatment when total CD4+ and CD8+ populations were examined (Supplementary Table S1). When assessed within TEM (the predominate circulating subset and a subset potentially relevant in tissue migration), a significant increase in CD162 expression was seen in both CD4+ and CD8 +TEM (Supplementary Table S1).

### Similar counts but altered functional responses of innate immune cells on FTY treatment

The numbers of circulating CD14+ monocytes and DCs were not altered by FTY treatment (Table 2). While the frequency of TNFα-expressing monocytes reduced by approximately 30% (*p* = 0.0441, data not shown), levels of IL-6 expression were unchanged (Supplementary Table S1). The expression of HLA-DR and CD86 on monocytes was unchanged, while DC expression of CD86 increased on-treatment (Supplementary Table S1).

### Pre-treatment CD4 + TCM may be higher in patients exhibiting disease activity on FTY

**Figure 5:**
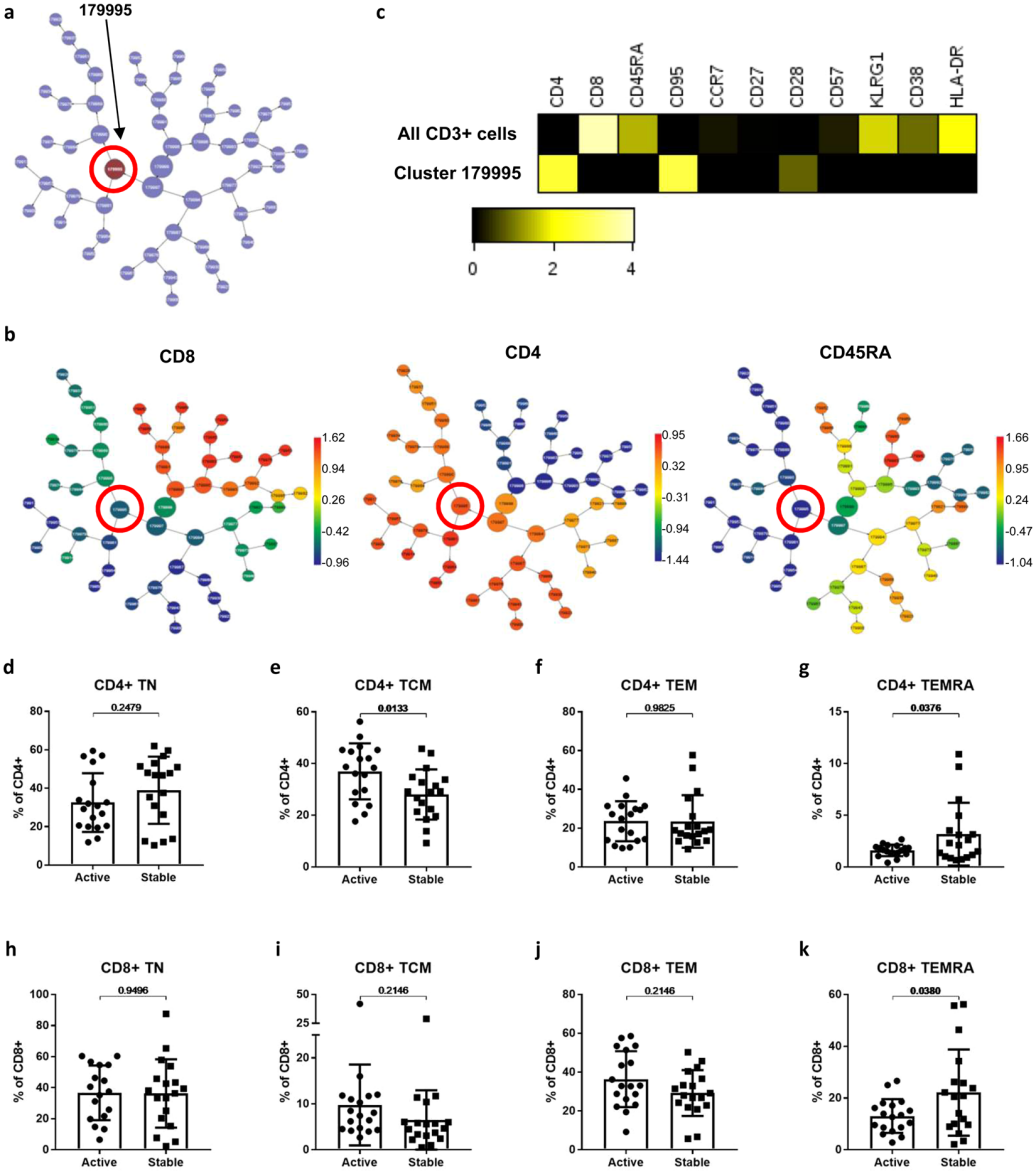
Pre-treatment differences in naive and memory T-cell subsets of patients with subsequently Active versus Stable disease On-treatment. Results from a CITRUS unbiased correlative analysis of pre-treatment samples comparing Active versus Stable cohorts are shown. Correlations between pre-treatment immune cell cluster abundances and on-treatment disease activity cohort were assessed using the significance analysis of microarrays (SAM) method with a false discovery rate of <1%. The cluster found to be significantly different in abundance between groups is shown in the CITRUS cluster tree (**A**). The cluster tree is shown coloured by level of expression of CD8, CD4 and CD45RA (**B**), where the colour scale bars indicate the transformed intensity of marker expression per cluster. A heatmap comparing the significant cluster with all CD3 + T cells is shown (**C**) and displays a transformed ratio of median marker expression using the lower of cluster and all CD3 + cells as the reference for each marker. The frequencies of traditionally analysed CD4 + and CD8 + naïve and memory T cells subsets in Active versus Stable cohort pre-treatment samples were compared using unpaired *t*-tests (**D–G**).

We examined correlations between on-treatment disease activity and pre-treatment T-cell profiles using the CITRUS (cluster identification, characterization, and regression) algorithm (Cytobank). Modelling at a false discovery rate of <1%, a cluster of cells (cluster 179995) was found to be higher in abundance in the pre-treatment samples of the subsequently Active versus the Stable cohort (Fig. 5A). Expression of key markers showed this cluster to consist of CD4+ memory cells (CD8-CD4+ CD45RA-; Fig. 5B). Compared with mean levels amongst all CD3+ cells, cluster 179995 cells exhibited lower expression of the senescence markers KLRG1 and CD57, higher expression of CD95 and the co-stimulatory molecule CD28, lower expression of activation markers HLA-DR and CD38, and a similar expression of CCR7 (Fig. 5C). Relatively high CD28 expression and low senescence marker expression suggests these memory cells did not represent a terminally differentiated population. The finding of a statistically significant between-group difference in a CD4+ memory cell cluster was verified on three independent replicates of the significance analysis of microarrays (SAM) analysis.

Additionally, we explored pre-treatment differences between Active and Stable cohorts using the manually gated immune cell subsets already examined longitudinally. We examined naïve and memory subsets (Fig. 5D–K) and found a higher frequency of CD4 + TCM (37% vs 28%, *p* = 0.0133) in the Active cohort, which may correspond with the cluster identified by the CITRUS analysis. Both CD4 + TEMRA (1.6% vs. 3.2%, *p* = 0.0376) and CD8 + TEMRA (13% vs. 22%, *p* = 0.038) appeared less abundant in the Active versus Stable cohort pre-treatment. No other immune cell subset frequencies analysed were found to be significantly different between the Active and Stable cohorts (Supplementary Table S2). Supplementary Table S3 shows absolute counts for all immune subsets compared between the cohorts; no significant differences in subset counts were found.

## Discussion

In this prospective study we examined FTY’s effects on a range of immune cell subsets and explored potential immune biomarkers of treatment response.

We focused on T-cell subsets previously implicated in MS pathogenesis. FTY induced decreases in almost all circulating T-cell subsets, indicating that they either recirculate to LNs and require S1P signals for LN egress or are peripherally depleted through other effects of FTY. It is well established that CCR7-expressing TN and TCM are most affected by FTY^4^. We also found decreases in CCR7- TEM counts on-treatment, in contrast to some studies^4,12^ and in keeping with others^15,16^. Both no change^12^ or a significant decrease^17^ in RTE frequency have previously been described. We found a reduction in both RTE numbers and their frequency on FTY. While limited by reliance on a typical surface-marker RTE definition, our results are consistent with FTY’s ability to inhibit thymic egress, as shown in murine studies^18^.

Senescent T cells were relatively spared on FTY resulting in an increase in their circulating frequencies. Senescent CD8 + T cells have low proliferative potential and may display enhanced cytotoxicity or suppressive functions, while CD28-CD4 + T cells have a Th1 phenotype^19^. An increase in these CD28- T-cell populations, termed premature immunosenescence, has been implicated in the pathogenesis of autoimmune disease^20,21^. T-cell senescence can be driven by homeostatic proliferation, which is increased in FTY-treated mice^22^. The relative increase in senescent T cells in our FTY-treated patients may therefore reflect less S1P-dependant trafficking of these subsets and/or a homeostatic response to immune cell sequestration.

Exhausted CD8+ T cells display low proliferative potential, upregulate inhibitory receptors and are unresponsive to antigenic stimulation^23^. Induction of exhaustion is therefore a potential therapeutic strategy in autoimmune disease^24^. We have found a relative increase in exhausted T cells on-treatment with FTY that may form part of its mode of action.

Circulating T cells on-treatment demonstrated an increase in expression of molecules important for CNS migration, a finding which was not entirely explained by changes in naïve and memory subset frequencies. The ability of circulating T cells to migrate into the CNS may be relevant to persistent disease activity on FTY, although our Active and Stable cohorts did not show differential expression of these molecules.

While reductions in total lymphocyte, T-cell and B-cell counts with FTY treatment have been well documented^2,4^, the effects of FTY on monocytes and DCs are less well known. In keeping with prior work^25,26^ we observed stable monocyte counts and increased frequencies of monocytes within PBMC due to lymphopenia. We further report that DC numbers were stable, such that DC frequencies similarly increased on-treatment. Functional responses of monocytes and DC may be altered by FTY. Consistent with a previous study^25^ we found a reduction in monocyte expression of pro-inflammatory TNFα and an increase in DC expression of CD86. Higher CD86 expression on antigen presenting cells has been associated with induction of tolerogenic Th2 responses^27,28^, and may be relevant to FTY’s therapeutic action.

A strength of this study is the identification, within a larger prospectively followed cohort, of patients either manifesting new disease activity (‘Active’) or not (‘Stable’) over approximately 2 years of treatment. Matching efforts ensured that the subgroups differed in disease activity on-treatment, but not obviously pre-treatment. A higher male-to-female ratio was found in our Active cohort, as was also seen in a previous study^15^; it is interesting to speculate whether this may reflect the known influence of sex on human immune responses^29^.

An unbiased CITRUS analysis identified a differential abundance of a CD4+ memory T-cell cluster between the cohorts pre-treatment, while traditional analysis suggested this difference lay primarily in CD4+ TCM. Th17 cells, often implicated in MS pathogenesis, are found predominantly within TCM^30^. Studying 23 FTY-treated MS patients, Song and colleagues^15^ found a higher frequency of CD4+ TCM in what they defined as ‘non-responders’ after 3–6 months of therapy, while the difference in TCM frequencies between our groups did not persist on-treatment (data not shown). We also describe a novel association between lower pre-treatment CD8+ and CD4+ TEMRA frequencies and on-treatment disease activity. The TEMRA phenotype can be associated with enhanced cytotoxicity, reduced functional capacity or suppressive functions^31^ and therefore functional profiling of these cells would enhance future studies.

A higher pre-treatment RTE frequency in suboptimal versus good responders to FTY has been described^32^ but was not replicated here. Our study included higher numbers of Active patients (18 versus 8) selected using different criteria and also defined RTE differently. Moreno-Torres and colleagues^33^ reported a higher pre-treatment CD56^bright^ NK cell frequency in patients who were subsequently stable versus active on FTY; a finding not replicated here. A cross-sectional study found higher CD56+ T-cell frequencies in FTY-treated versus untreated patients, and an even higher frequency of these cells during relapse in 4 patients^34^. We find CD56-expressing T-cell counts unaffected by FTY and confirm the resulting treatment-induced increase in their frequency. There was no difference in CD56+ T-cell frequency between Active and Stable cohorts, although we chose to avoid peri-relapse samples, and hence cannot assess the association of particular subsets with episodic disease activity. Together, these findings suggest that any relapse-associated increase in CD56+ T cells may be transient.

There are limitations to our study. While our Active and Stable cohorts exhibited different degrees of on-treatment clinical and MRI disease activity, we note that on-treatment activity is not equivalent to treatment ‘non-response’, since even Active patients may have experienced a reduction in disease activity with FTY. Conversely, stability during a 2-year follow-up could reflect an individual’s natural history rather than a treatment effect. Though clinical and MRI data was longitudinally collected, immune profiling was performed at a single on-treatment time point. The assessment of between-group differences using the unbiased CITRUS algorithm was restricted to a correlative, hypothesis-generating analysis due to limited numbers. This analysis can include only markers measured simultaneously, thus we limited our use to a single panel of markers which included multiple phenotypic and functional markers of distinct T-cell subsets. Future biomarker discovery studies could include more extensive marker panels covering multiple immune cell types and their functional molecules in similar analyses by using mass cytometry, which allows for many more markers to be measured simultaneously, or by utilising alternative cell phenotyping approaches such as single-cell sequencing. This would allow assessment of treatment effects and potential predictive utility of the balance between subsets of T cells, B cells and myeloid cells considered increasingly relevant to defining the state of MS disease activity^35,36^. Our study involved multiple comparisons, which carries a risk of false positive discoveries. Therefore, potential biomarkers of FTY response identified here require validation in an independent cohort.

In summary, in this prospective and rigorously studied cohort, we document the effects of FTY on a range of peripheral immune cell subsets, including several not previously studied, with findings relevant to our understanding of immune cell trafficking and potential mechanisms of action of FTY. Combining traditional flow cytometry analysis and an exploratory unbiased multiparametric analysis, we find that pre-treatment T-cell subset frequencies, particularly those of CD4 + TCM cells, may have value in predicting FTY treatment response and warrant further study.

## Patients and Methods

### Study design

Patients enrolled in this study were recruited in the Canadian prospective multicentre observational treatment study of FTY (ClincalTrialGov ID:NCT02137707). The overall study included RRMS patients deemed by their treating physicians as suitable for FTY, aged 18–65 years with an EDSS of ≤7.0. Participants were followed clinically, radiologically and with periodic blood sampling (Supplementary Fig. S1). All participants provided written informed consent. The study was approved by and carried out in accordance with the guidelines and regulations of the McGill University Health Center (MUHC) research ethics board (REB) panel (2013-173: NEU-12-016 (IGLOO)).

Of the 135 participants in the overall study, 36 were selected for this study. ‘Active’ (n = 18) and ‘Stable’ (n = 18) cohorts were selected based on the development or absence of on-treatment relapses and/or NEL on follow-up brain MRIs, as outlined in Supplementary Fig. S4. Pre-treatment and on-treatment PBMC samples were analysed for each patient. Samples failing pre-defined quality control (QC) criteria and those obtained within 1 month following intravenous steroid therapy or 1 month before or following a clinical relapse were excluded. On-treatment samples from 1-year on-treatment were used for most patients (n = 22). When the 1-year samples were not usable due to either peri-sampling relapse activity, missing samples or samples failing our pre-defined QC criteria, we instead used 6-month (n = 12) or 2-year (n = 2) on-treatment samples. Of note, the timepoints selected for the on-treatment samples were balanced between Active and Stable subgroups.

### Clinical relapse criteria

A clinical relapse was defined as new, worsening or recurrent neurological symptoms that occurred at least 30 days after the onset of a preceding relapse and lasted at least 24 hours without fever or infection. Relapses were confirmed by the treating neurologist as being accompanied by an increase of at least 0.5 points on the overall EDSS, at least 1 point on two functional systems of the EDSS or at least 2 points on one functional system (excluding bowel and bladder and cerebral function systems).

### MRI acquisition

Standardised serial brain MRIs were acquired pre-treatment (all patients) and at month 12 (n = 27) and month 24 (n = 29) on-treatment on a 3 T Siemens TIM Trio MRI scanner (Siemens Healthcare, Erlangen, Germany). Serial scans from each individual were performed consistently using the same receiver coil. Axial 2D, T2-weighted images were acquired using a turbo spin-echo (TSE) sequence with TR = 4500 ms, TE = 83 ms, turbo factor = 11, in-plane voxel dimensions of 1 × 1 mm2 and slice thickness = 3 mm; Axial 2D, proton density-weighted images were acquired using a TSE sequence with TR = 2200 ms, TE = 10 ms, turbo factor = 4, in-plane voxel dimensions of 1 × 1 mm^2^ and slice thickness = 3 mm; Axially-acquired 3D, T1-weighted images were acquired using a 3D, fast, low-angle shot (FLASH) sequence with TR = 28 ms, TE = 6.15 ms, flip angle = 27 degrees, in-plane voxel dimensions of 1 × 1 mm2 and slice partition thickness = 3 mm; Sagittal 3D fluid-attenuated inversion recovery (FLAIR) images were acquired using an inversion-prepared variable flip angle TSE sequence with TI = 2200 ms, TR = 6 s, TE = 355 ms, turbo factor = 141, isotropic 1 mm3 voxels and GRAPPA acceleration factor R = 2 in the first phase encode direction.

### Measurement of new or enlarging T2 lesions

New/enlarging T2 lesions were detected on follow-up scans with respect to reference scans using a two-stage process as previously described^37^. Briefly, the first stage performs a voxel-by-voxel tissue classification and identifies potential new/enlarging T2 lesions using a Bayesian classifier that considers MRI intensities in the follow-up scan, intensity differences between co-registered reference and follow-up scans and atlas-based templates for healthy tissue and T2 hyperintense lesions. The second stage groups voxels identified as new/enlarging T2 lesions in the first classification stage to form a set of new/enlarging T2 lesions candidates, which are further classified using a random forest classifier based on lesion-level features.

### Total lymphocyte counts and PBMC processing

Complete blood counts including TLC were serially performed by the certified clinical laboratory of the recruiting hospital for each patient. PBMC were isolated from whole blood (Ficoll density centrifugation; GE Healthcare) and cryopreserved using rigorous standardized operating procedures (SOPs) developed and validated by our Experimental Therapeutics Program for all steps of sample procurement, processing, cryopreservation, storage and subsequent thawing. Following our strict SOPs for PBMC isolation and cryopreservation^36^, the majority of thawed samples had viabilities of over 90%. Any sample with cell viability of less than 70% was excluded from analysis.

### PBMC staining

Immune cell subset phenotypic and functional analyses within PBMC were performed in batch by a blinded operator. Each batch included the pre- and on-treatment PBMC samples from matched pairs of Active and Stable patients as well as an aliquot of an internal control sample used across batches. Staining combinations and reagent details are provided in Supplementary Tables S4 and S5.

Thawed PBMC were suspended in X-VIVO 10 serum-free cell medium (Lonza) and rested at 37 degrees Celsius (5% CO2) for four hours. For T-cell intracellular cytokine staining (ICS), phorbol 12-myristate 13-acetate (PMA; 20 ng/ml; Sigma-Aldrich), ionomycin (1 µg/ml; Sigma-Aldrich) and GolgiStop (Monensin; BD Biosciences) were added to PBMC. For monocyte ICS, lipopolysaccharide (1 µg/ml; Sigma-Aldrich) and GolgiStop were added. Prior to cell surface staining, rested or stimulated cells were stained with a viability dye as per manufacturer recommendations (Live/Dead fixable aqua dead cell stain; Invitrogen). Briefly, cells were washed in cold phosphate-buffered saline (PBS) and resuspended in cold PBS containing freshly reconstituted fluorescent reactive dye. Samples were incubated on ice for 30 minutes protected from light and then washed in cold PBS.

Cell surface marker staining was performed prior to intracellular staining. Cells were processed and stained as per strict SOPs developed from published protocols^38^ and reagent manufacturer recommendations (BD Biosciences and eBioscience). Briefly, surface staining was performed using flow cytometry (FACS) buffer (PBS containing 1% FCS) or Brilliant buffer (BD Biosciences) depending on the specific antibody-fluorochrome combinations used. Cells were suspended in buffer containing pre-mixed combinations of fluorochrome-conjugated antibodies and incubated at either room temperature for 30 minutes (Brilliant buffer) or on ice for 20 minutes (FACS buffer). Cells were washed in FACS buffer and then permeabilized and fixed using either Cytofix/Cytoperm (BD Biosciences) or Intracellular Fixation Buffer (eBioscience). Following fixation/permeabilization, intracellular staining was performed using pre-mixed fluorochrome-antibody combinations in an appropriate permeabilization buffer and samples were incubated for 30 minutes. The cells were washed and resuspended in FACS buffer for flow cytometric analysis. Unstained controls, isotype controls and internal controls were processed alongside experimental samples.

### Flow cytometry and analysis

Acquisition and analysis were carried out by a blinded operator using an LSR Fortessa flow cytometer (BD Biosciences). Manual two-dimensional gating was performed using FlowJo (Tree Star). Representative examples of staining and gating of major immune cell and T-cell subsets are provided in Supplementary Figs. S5 and S6. Absolute counts of lymphocyte and monocyte subsets were estimated using the fraction of live singlet PBMC within the gate of interest multiplied by the sum of the total lymphocyte and monocyte counts as performed by the clinical laboratory on the same blood draw. Pre- and on-treatment measures were compared using two-tailed paired *t*-tests; *p* values are presented both unadjusted and following Bonferroni correction for multiple comparisons and considered statistically significant at <0.05. Active and Stable cohorts were compared using two-tailed unpaired *t*-tests; unadjusted *p* values are displayed for this analysis given its extensive and exploratory nature. Data were visualized using heatmaps and viSNE (Cytobank)^39^.

Correlations between immune cell subset measures and on-treatment disease activity cohort (Active vs. Stable) were assessed using the CITRUS (Cytobank). CITRUS automates discovery of stratifying biological signatures amongst samples with a known clinical endpoint^40^. Manual gating of PBMC, live cells and total CD3 + cells was first performed in Cytobank as per the traditional analysis gating strategy (Supplementary Figs. S5 and S6) for all pre-treatment samples stained with the naïve/memory/senescent (NMS) T-cell panel (Supplementary Table S4). Unsupervised hierarchical clustering was performed gated on total live CD3 + cells using equal sampling of 9800 events from each sample. Minimum cluster size was set to 3%. Markers used for clustering were CD4, CD8, CD45RA, CD28, CD27, CD57 and KLRG1. The relative abundance of each cellular cluster was calculated for each sample. Associations between disease activity cohort and cluster abundances were identified using the significance analysis of microarrays (SAM) method with a false discovery rate of <1% and cross validation fold number of 5. The analysis was repeated three times with identical parameters to ensure reproducibility of the results. Heatmaps were generated comparing marker expression within the cellular cluster of interest versus all CD3 + T cells, displayed as a transformed ratio of median marker expression using the lower of cluster and all CD3 + cells as the reference for each marker.

## Supplementary information

Supplementary information.

## Data Availability

The datasets generated during and/or analysed during the current study are available from the corresponding author on reasonable request.
