# Supplementary material for: Pre-treatment T-cell subsets associate with fingolimod treatment responsiveness in multiple sclerosis
Source: Sci Rep. 2020 Jan 15;10:356. doi: 10.1038/s41598-019-57114-2 (PMC6962338; doi:10.1038/s41598-019-57114-2)
Supplement: Supplementary file 1 — Supplementary information. [file 41598_2019_57114_MOESM1_ESM.pdf]

Pre-treatment T-cell subsets associate with fingolimod treatment responsiveness in multiple sclerosis

Mahtab Ghadiri, Ayman Rezk, Rui Li, Ashley Evans, Paul S Giacomini, Michael H Barnett, Jack Antel, Amit Bar-Or

Supplementary Information

Supplementary Figure S1. Changes in additional T-cell subset frequencies On-treatment versus Pre-treatment with FTY.

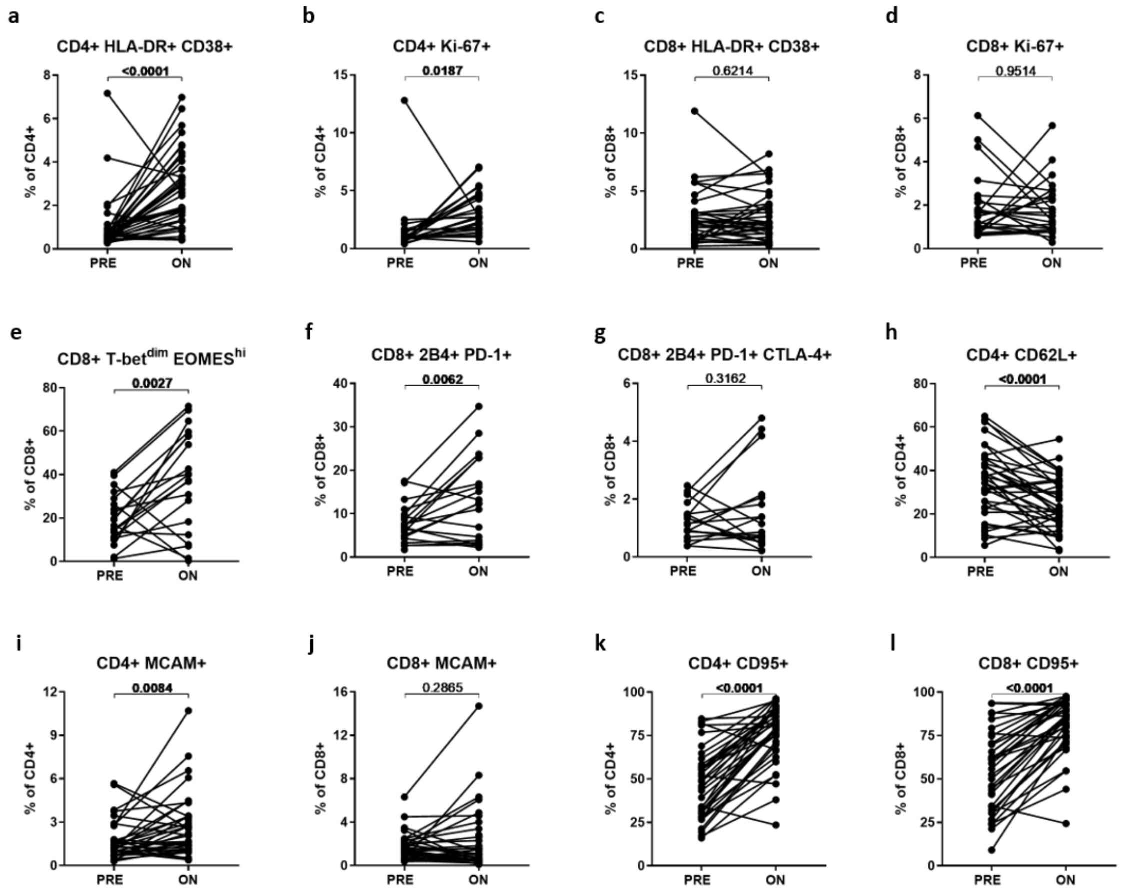

Figure S1. Frequencies, within total CD4+ or CD8+ T cells, of potentially MS relevant T-cell subsets were compared in pre- and on-treatment samples using paired *t*-tests (A-L).

Supplementary Figure S2. Changes in Treg subset frequencies On-treatment versus Pre-treatment with FTY.

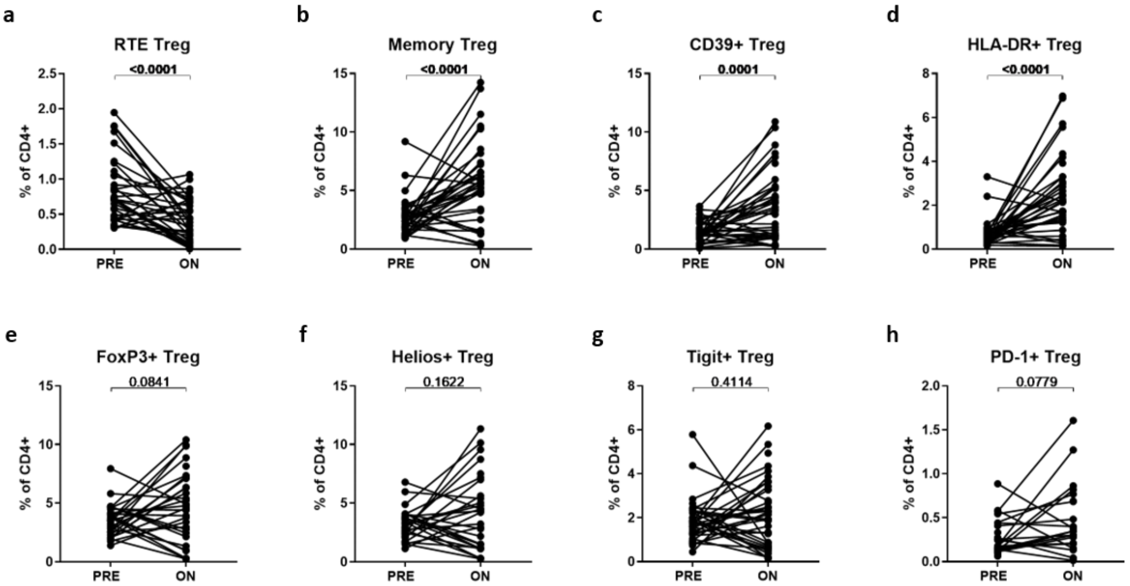

Figure S2. Frequencies, within total CD4+ T cells, of Treg subsets were compared in pre- and on-treatment samples using paired *t*-tests (A-H).

Supplementary Figure S3. Canadian prospective multicenter observational treatment study of fingolimod: Study schedule.

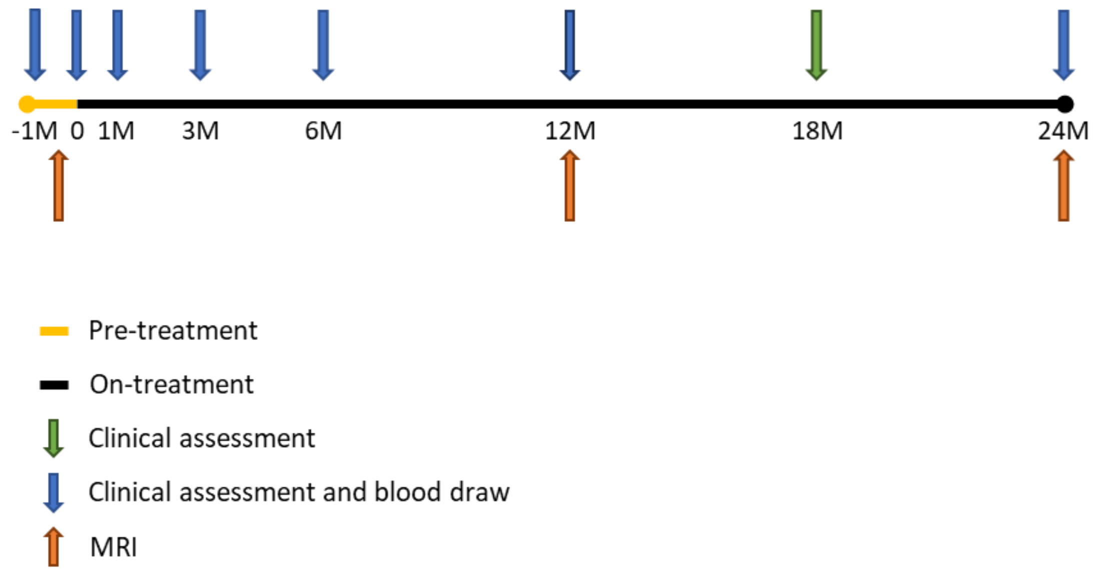

Figure S3. The study schedule for the Canadian prospective multicenter observational treatment study of fingolimod (ClincalTrialGov ID:NCT02137707) is shown, including the timing of pre-treatment and on-treatment clinical assessments, blood draws and MRIs.

Supplementary Figure S4. Patient selection.

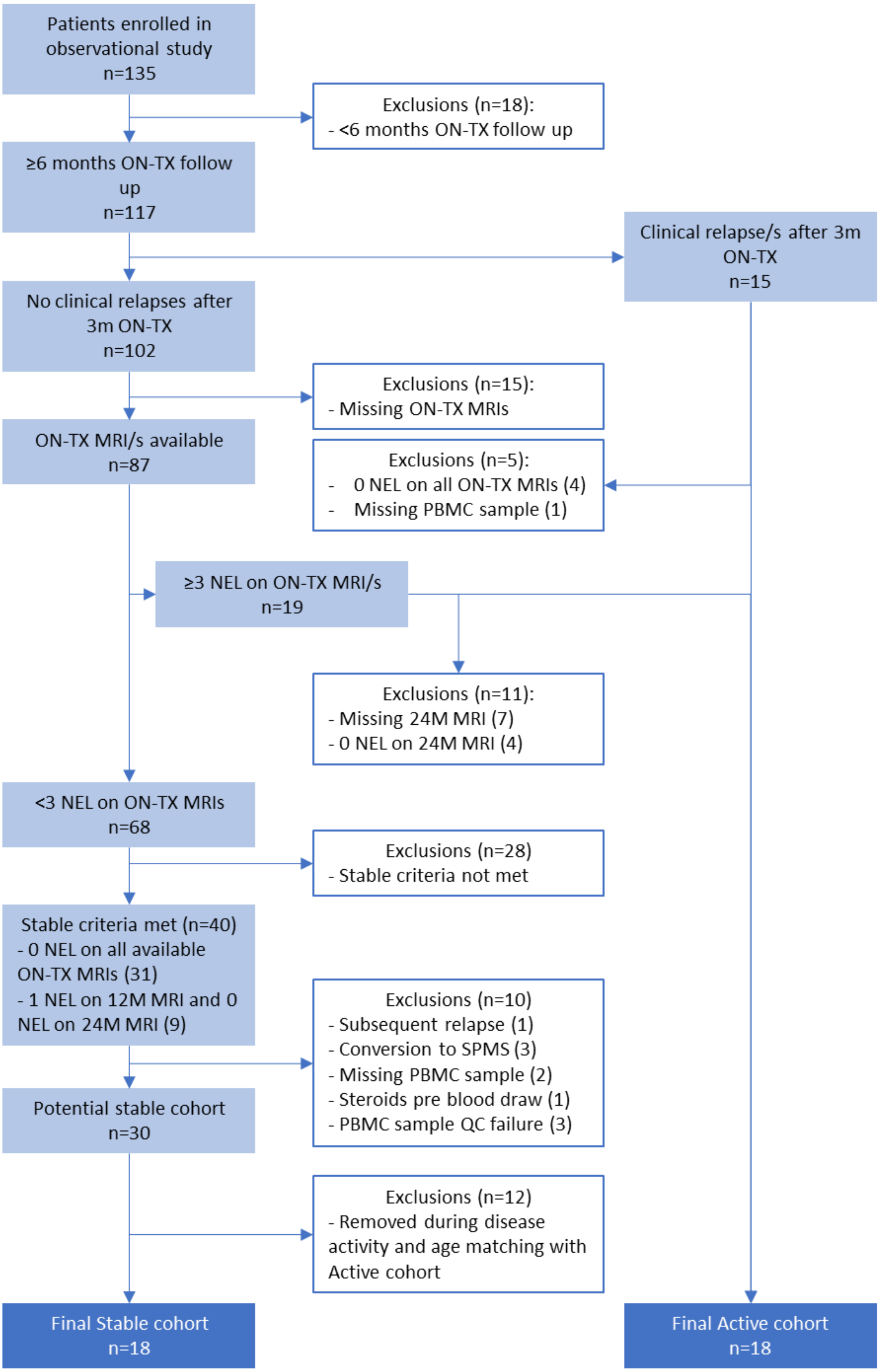

Figure S4. Patient selection from the Canadian prospective multicenter observational treatment study of fingolimod for the Active and Stable cohorts of the current immune monitoring study is outlined in the flowchart.

Supplementary Figure S5. Early gating and major immune cell subset gating strategy.

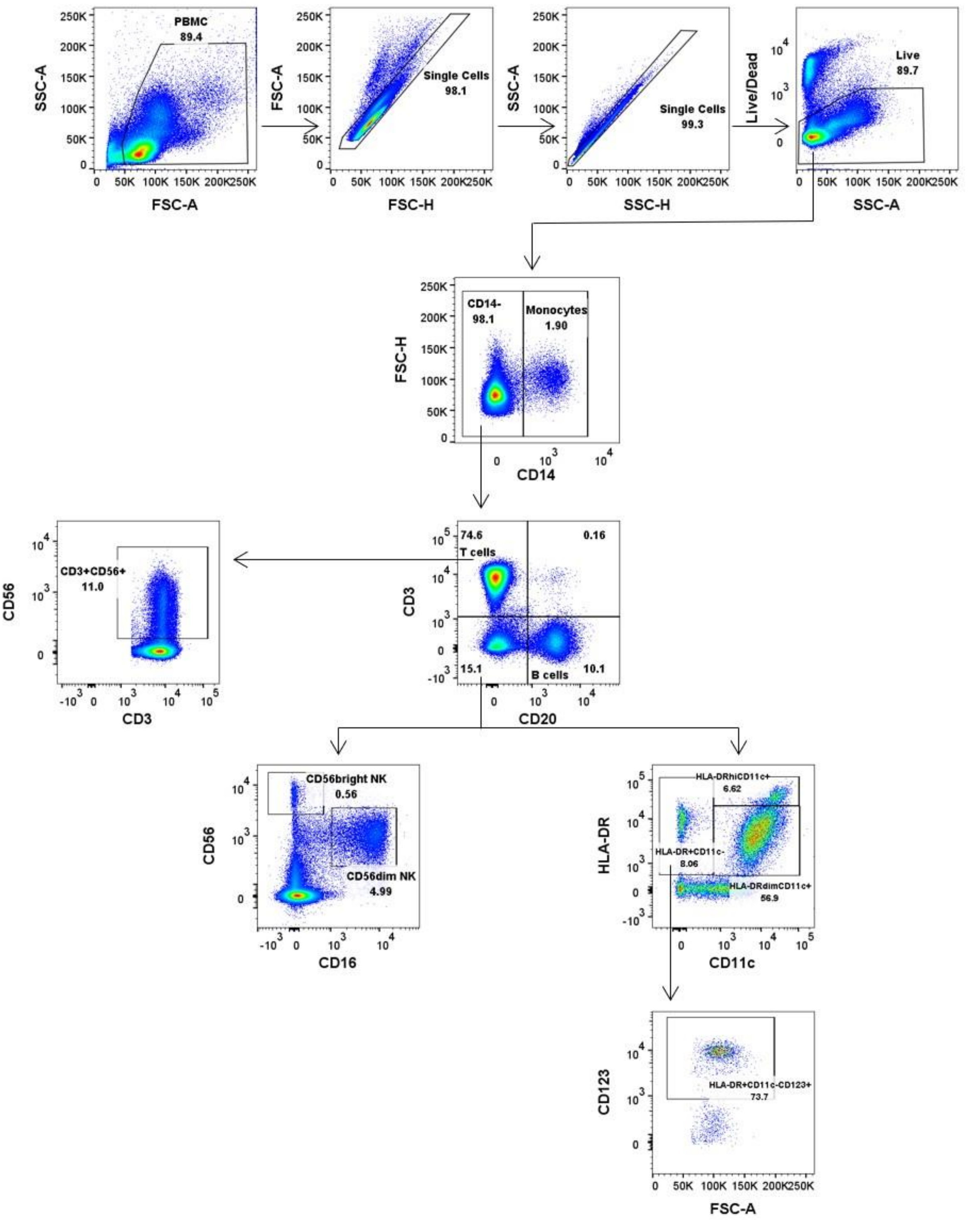

Figure S5. The early flow cytometry gating strategy, for exclusion of debris, doublets and dead cells, followed by the gating strategy for major immune cell subsets within PBMC, is shown.

Supplementary Figure S6. T-cell subset gating strategy.

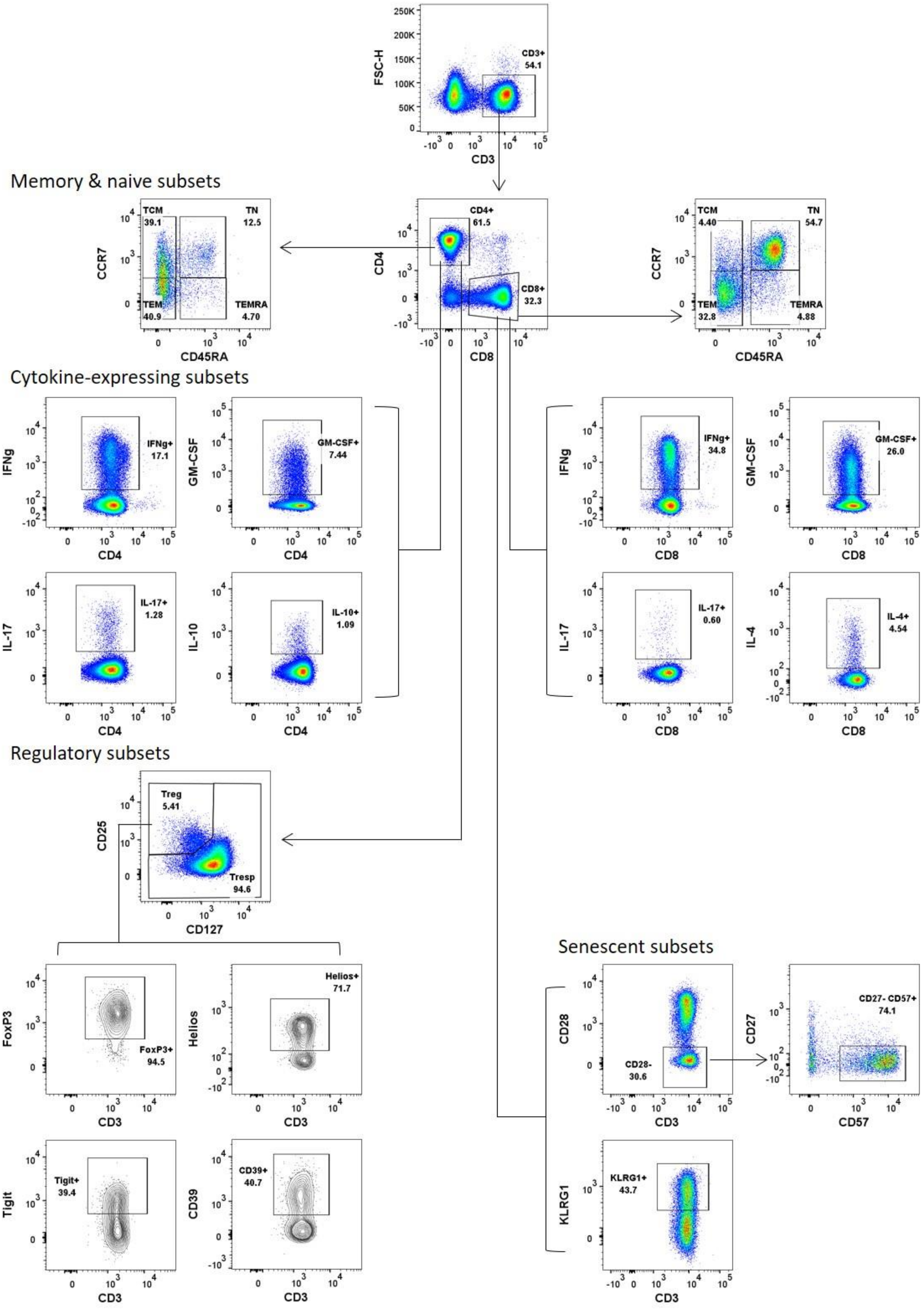

Figure S6. The gating strategy for a range of T-cell subsets is shown. Gating is shown beginning from live, singlet PBMC.

Supplementary Table S1. Changes in surface molecule expression On-treatment versus Pre-treatment with FTY.

Cell subset	Marker	Pre MFI (SD)	On MFI (SD)	Change (On – Pre)	*p* value (unadjusted)	*p* value (adjusted)
CD4+ T cells	CD11a	3738 (823)	5716 (2161)	1977	<0.0001	<0.0021
	CD6	444 (112)	478 (144)	34	0.0228	ns
	CD162	4769 (2738)	7068 (4685)	2299	0.0003	0.0063
	CD49d	649 (293)	886 (638)	237	0.0077	ns
CD8+ T cells	CD11a	4934 (1558)	7575 (2160)	2641	<0.0001	<0.0021
	CD6	262 (68)	228 (82)	-34	0.0003	0.0063
	CD162	6942 (3514)	9024 (5010)	2082	0.0006	0.0126
	CD49d	845 (438)	1229 (554)	384	<0.0001	<0.0021
CD4+ TEM	CD11a	5677 (1049)	6511 (2367)	833	0.0302	ns
	CD6	424 (114)	443 (150)	20	ns	ns
	CD162	6117 (3209)	8372 (5639)	2255	0.0010	0.0210
	CD49d	884 (464)	808 (723)	-235	ns	ns
CD8+ TEM	CD11a	8473 (1702)	9020 (2058)	547	ns	ns
	CD6	256 (76)	272 (107)	16	ns	ns
	CD162	8309 (3973)	10010 (5623)	1701	0.0012	0.0252
	CD49d	1311 (572)	1459 (599)	149	0.0261	ns
Monocytes	IL-6	3777 (2789)	2651 (1386)	-1126	ns	ns
	HLA-DR	8300 (3099)	8867 (3037)	567	ns	ns
	CD86	1454 (404)	1514 (487)	59	ns	ns
DCs	HLA-DR	5664 (2202)	5376 (1495)	-288	ns	ns
	CD86	696 (265)	854 (276)	158	0.0017	0.0357

Mean fluorescence intensity (MFI) and standard deviation (SD) of surface molecules pre-treatment (Pre) and on-treatment (On) are shown (n=10-35). The differences between on-treatment and pre-treatment MFIs are shown. The results of paired t-tests comparing the two timepoints are shown both unadjusted and after a Bonferroni correction for multiple comparisons. ns = not significant.

Supplementary Table S2. Pre-treatment cell subset frequencies in subsequently Active versus Stable cohorts.

	Cell subset	Within population	Active mean % (SD)	Stable mean % (SD)	*p* value
**Major T-cell subsets**	Total CD3+	PBMC	59 (11)	61 (17)	ns
	CD4+	CD3+	68 (10)	63 (18)	ns
	CD8+	CD3+	25 (10)	29 (14)	ns
**Naïve & memory T-cell subsets**	CD4+ TN	CD4+	33 (15)	39 (17)	ns
	CD4+ TCM	CD4+	37 (11)	28 (10)	0.0133
	CD4+ TEM	CD4+	24 (10)	23 (14)	ns
	CD4+ TEMRA	CD4+	1.6 (0.6)	3.1 (3.0)	0.0376
	CD8+ TN	CD8+	35 (18)	36 (22)	ns
	CD8+ TCM	CD8+	10 (9)	7 (6)	ns
	CD8+ TEM	CD8+	36 (14)	29 (12)	ns
	CD8+ TEMRA	CD8+	13 (7)	22 (17)	0.0380
**Cytokine-expressing T-cell subsets**	CD4+ IFNγ+	CD4+	14 (7)	13 (7)	ns
	CD4+ GM-CSF+	CD4+	12 (5)	12 (6)	ns
	CD4+ IL-17+	CD4+	0.8 (0.4)	0.7 (0.3)	ns
	CD4+ IL-22+	CD4+	1.2 (0.8)	1.1 (0.5)	ns
	CD4+ IL-4+	CD4+	2.9 (1.5)	3.3 (1.6)	ns
	CD4+ IL-10+	CD4+	1.1 (1.0)	0.7 (0.6)	ns
	CD8+ IFNγ+	CD8+	36 (17)	42 (18)	ns
	CD8+ GM-CSF+	CD8+	10 (6)	9 (4)	ns
	CD8+ IL-17+	CD8+	0.5 (0.3)	0.7 (0.8)	ns
	CD8+ IL-4+	CD8+	3.0 (3.5)	2.9 (2.0)	ns
**Regulatory T-cell subsets**	Total Treg	CD4+	4.0 (1.2)	4.4 (2.1)	ns
	FoxP3+ Treg	CD4+	3.4 (1.0)	3.6 (1.7)	ns
	RTE Treg	CD4+	0.9 (0.5)	0.7 (0.4)	ns
	Memory Treg	CD4+	2.3 (0.8)	3.0 (2.1)	ns
	CD39+ Treg	CD4+	1.4 (0.8)	1.5 (1.0)	ns
	Helios+ Treg	CD4+	3.0 (1.0)	3.2 (1.6)	ns
	Tigit+ Treg	CD4+	1.7 (0.6)	1.9 (1.3)	ns
	HLA-DR+ Treg	CD4+	0.6 (0.3)	0.8 (0.8)	ns
	PD-1+ Treg	CD4+	0.2 (0.2)	0.3 (0.2)	ns
**Senescent T-cell subsets**	CD8+ CD28- CD27- CD57+	CD8+	17 (15)	22 (15)	ns
	CD8+ KLRG1+	CD8+	32 (19)	32 (20)	ns
	CD4+ CD28-	CD4+	1.9 (3.2)	2.3 (3.5)	ns

	Cell subset	Within population	Active mean % (SD)	Stable mean % (SD)	*p* value
**Activated & Proliferating T-cell subsets**	CD4+ HLA-DR+ CD38+	CD4+	0.7 (0.4)	1.3 (1.7)	ns
	CD4+ Ki67+	CD4+	1.2 (0.7)	1.7 (3.0)	ns
	CD8+ HLA-DR+ CD38+	CD8+	2.3 (1.6)	2.9 (2.9)	ns
	CD8+ Ki67+	CD8+	1.7 (1.4)	1.9 (1.4)	ns
**Exhausted T-cell subsets**	CD8+ $Tbet^{dim}$ $EOMES^{hi}$	CD8+	22 (14)	17 (10)	ns
	CD8+ PD-1+ 2B4+	CD8+	7 (4)	10 (8)	ns
	CD8+ PD-1+ 2B4+ CTLA-4+	CD8+	1.2 (0.5)	1.6 (1.4)	ns
**T-cells expressing other markers of interest**	CD4+ MCAM+	CD4+	1.8 (1.4)	1.6 (1.4)	ns
	CD8+ MCAM+	CD4+	2.0 (1.6)	1.5 (0.7)	ns
	CD4+ RTE	CD4+	30 (16)	39 (17)	ns
	CD4+ CD62L+	CD4+	31 (16)	37 (16)	ns
	CD4+ CD95+	CD4+	50 (19)	45 (21)	ns
	CD8+ CD95+	CD8+	55 (22)	53 (24)	ns
	CD3+ CD56+	PBMC	4.8 (6.2)	4.5 (4.8)	ns
	CD4+ CD56+	CD4+	1.1 (2.4)	1.1 (1.7)	ns
**Other cell types**	Total B cells	PBMC	13 (7)	10 (5)	ns
	Total NK cells	PBMC	5.1 (3.3)	6.1 (6.7)	ns
	$CD56^{bright}$ NK	PBMC	0.6 (0.3)	0.9 (0.8)	ns
	$CD56^{dim}$ NK	PBMC	3.2 (1.9)	4.1 (5.2)	ns
	Monocytes	PBMC	5.5 (4.7)	6.5 (3.7)	ns
	Total DC	PBMC	8.6 (6.9)	7.7 (5.1)	ns
	HLA-DR+ CD11c-CD123+ DC	PBMC	0.6 (0.3)	0.8 (0.6)	ns
	$HLA\text{-}DR^{hi}$ CD11c+ DC	PBMC	0.9 (0.8)	0.9 (0.6)	ns
	$HLA\text{-}DR^{dim}$ CD11c+ DC	PBMC	5.4 (4.9)	5.4 (4.3)	ns

Mean and standard deviation (SD) of all immune cell subset frequencies are shown for Active and Stable cohorts at the pre-treatment timepoint (n=22-36). Frequencies within the stated population ('Within population' column) were analysed. Immune cell subset frequencies of the Active and Stable cohorts were compared using unpaired t-tests. ns = not significant.

Supplementary Table S3. Pre-treatment cell subset counts in subsequently Active versus Stable cohorts.

	Cell subset	Active mean (SD)	Stable mean (SD)	*p* value
**TLC**	TLC	2224 (958)	1845 (808)	ns
**Major T-cell subsets**	Total CD3+	1639 (680)	1559 (719)	ns
	CD4+	1128 (473)	1050 (492)	ns
	CD8+	413 (258)	422 (264)	ns
**Naïve & memory T-cell subsets**	CD4+ TN	384 (275)	428 (252)	ns
	CD4+ TCM	397 (170)	308 (194)	ns
	CD4+ TEM	268 (171)	222 (160)	ns
	CD4+ TEMRA	18 (11)	25 (25)	ns
	CD8+ TN	153 (127)	153 (117)	ns
	CD8+ TCM	49 (79)	31 (40)	ns
	CD8+ TEM	144 (90)	121 (84)	ns
	CD8+ TEMRA	47 (37)	92 (102)	ns
**Cytokine-expressing T-cell subsets**	CD4+ IFNγ+	154 (89)	122 (83)	ns
	CD4+ GM-CSF+	135 (69)	112 (70)	ns
	CD4+ IL-17+	7 (3)	6 (4)	ns
	CD4+ IL-22+	12 (6)	10 (7)	ns
	CD4+ IL-4+	28 (15)	31 (21)	ns
	CD4+ IL-10+	11 (11)	9 (7)	ns
	CD8+ IFNγ+	134 (93)	150 (102)	ns
	CD8+ GM-CSF+	46 (59)	38 (40)	ns
	CD8+ IL-17+	2 (1)	2 (1)	ns
	CD8+ IL-4+	16 (34)	14 (17)	ns
**Regulatory T-cell subsets**	Total Treg	48 (19)	43 (25)	ns
	FoxP3+ Treg	43 (17)	33 (21)	ns
	RTE Treg	11 (9)	9 (5)	ns
	Memory Treg	26 (8)	26 (19)	ns
	CD39+ Treg	17 (10)	14 (11)	ns
	Helios+ Treg	38 (15)	28 (17)	ns
	Tigit+ Treg	20 (6)	17 (11)	ns
	HLA-DR+ Treg	7 (4)	6 (4)	ns
	PD-1+ Treg	2 (1)	2 (2)	ns
**Senescent T-cell subsets**	CD8+ CD28- CD27- CD57+	50 (52)	98 (103)	ns
	CD8+ KLRG1+	109 (72)	136 (142)	ns
	CD4+ CD28-	15 (27)	21 (37)	ns

	Cell subset	Active mean (SD)	Stable mean (SD)	*p* value
**Activated & Proliferating T-cell subsets**	CD4+ HLA-DR+ CD38+	7 (4)	7 (5)	ns
	CD4+ Ki67+	16 (12)	15 (14)	ns
	CD8+ HLA-DR+ CD38+	9 (6)	12 (16)	ns
	CD8+ Ki67+	8 (5)	10 (11)	ns
**Exhausted T-cell subsets**	CD8+ $Tbet^{dim}$ $EOMES^{hi}$	116 (70)	85 (81)	ns
	CD8+ PD-1+ 2B4+	73 (74)	61 (34)	ns
	CD8+ PD-1+ 2B4+ CTLA-4+	7 (5)	7 (5)	ns
**T-cells expressing other markers of interest**	CD4+ MCAM+	21 (21)	13 (10)	ns
	CD8+ MCAM+	8 (5)	6 (4)	ns
	CD4+ RTE	353 (230)	399 (247)	ns
	CD4+ CD62L+	359 (239)	410 (327)	ns
	CD4+ CD95+	533 (260)	442 (283)	ns
	CD8+ CD95+	209 (131)	229 (197)	ns
	CD3+ CD56+	62 (42)	75 (102)	ns
	CD4+ CD56+	0 (0)	1 (3)	ns
**Other cell types**	Total B cells	407 (310)	245 (142)	ns
	Total NK cells	160 (130)	135 (125)	ns
	$CD56^{bright}$ NK	17 (9)	21 (21)	ns
	$CD56^{dim}$ NK	101 (87)	90 (100)	ns
	Monocytes	574 (225)	523 (120)	ns
	Total DC	262 (246)	158 (85)	ns
	HLA-DR+ CD11c-CD123+ DC	16 (7)	18 (11)	ns
	$HLA\text{-}DR^{hi}$ CD11c+ DC	27 (21)	20 (10)	ns
	$HLA\text{-}DR^{dim}$ CD11c+ DC	144 (107)	109 (73)	ns

Mean and standard deviation (SD) of absolute cell counts for all immune cell subsets are shown for Active and Stable cohorts at the pre-treatment timepoint (*n*=21-33). Immune cell subset counts of the Active and Stable cohorts were compared using unpaired *t*-tests, with no statistically significant differences seen between groups. ns = not significant.

Supplementary Table S4: Immune phenotyping panels.

PANEL	FITC	PERCP	APC	AF700	APC-CY7	BV421	BV510	BV650	BV786	BUV 395	BUV 661	PE	PE-TXR	PE-CY7
**NMS**	CD45-RA	CD3	CD95	CD8	KLRG1	CD28	L/D	CD27	CD4	CD38	HLA-DR	CD62L	CD57	CCR7
**RR**	Helios	TIGIT	CD39		CD45-RO	CD25	L/D	PD-1	CD4	CD3	HLA-DR	CD31	FoxP3	CD127
**CYTO**	CD45-RA	IL-22	IL-10	CD8	CD3	IL-17	L/D		CD4	IFNγ		GM-CSF	IL-4	CCR7
**ADH**	CD6	CD3	MCAM	CD11a	CD45-RO	CD49D	L/D	CD20	CD4	CD8		CD162	FoxP3	CCR7
**EXH**	CD45-RA	CCR7	Tbet	CD8		Ki-67	L/D	PD-1	CD4	CD3		CTLA4	2B4	EOMES
**BMND**		CD20	CD86	CD3	CD16	CD123	L/D	CD4	CD14		HLA-DR	CD11c	CD56	CD1c
**MCC**	CD14				CD16	IL-6	L/D			TNFα				

Antibody-fluorochrome combinations are listed for each staining panel: NMS = naïve/memory/senescent T-cell panel; RR = regulatory & recent thymic emigrant T-cell panel; CYTO = cytokine-expressing T-cell panel; ADH = adhesion molecule panel; EXH = exhaustion marker T-cell panel; BMND = B cell/monocyte/natural killer cell/dendritic cell panel; MCC = monocyte cytokine panel. L/D = live/dead viability staining. Fluorochromes listed or those with similar emission and excitation spectra were used as per the detailed list of flow cytometry antibodies Supplementary Table S4.

Supplementary Table S5: Staining reagents.

Antibody	Fluorochrome conjugation	Clone	Source
**CD3**	PerCP-Cy5.5	SK7	Biolegend
**CD3**	BUV395	SK7	BD Biosciences
**CD3**	APC-H7	SK7	BD Biosciences
**CD3**	APC-R700	UCHT1	BD Biosciences
**CD4**	BV786	SK3	BD Biosciences
**CD4**	BV650	SK3	BD Biosciences
**CD8**	AlexaFluor700	RPA-T8	BD Biosciences
**CD8**	BUV395	RPA-T8	BD Biosciences
**CD20**	BV650	2H7	BD Biosciences
**CD20**	PerCP	2H7	Biolegend
**CD45RA**	FITC	HI100	BD Biosciences
**CD45RO**	APC-H7	UCHL1	BD Biosciences
**CCR7**	PE-Cy7	3D12	BD Biosciences
**CCR7**	PerCP-Cy5.5	150503	BD Biosciences
**CD95**	APC	DX2	BD Biosciences
**CD28**	BV421	CD28.2	BD Biosciences
**CD27**	BV650	M-T271	BD Biosciences
**CD57**	PE-CF594	NK-1	BD Biosciences
**KLRG1**	APC-Cy7	REA261	Miltenyi Biotech
**CD38**	BUV395	HB7	BD Biosciences
**HLA-DR**	BUV661	G46-6	BD Biosciences
**CD62L**	PE	DREG-56	BD Biosciences
**CD25**	BV421	2A3	BD Biosciences
**CD127**	PC7	R34.34	Beckman Coulter
**FoxP3**	PE-CF594	236A/E7	BD Biosciences
**CD39**	APC	TU66	BD Biosciences
**Tigit**	PerCP-eFluor710	MBSA43	ebioscience
**Helios**	AlexaFluor488	22F6	Biolegend
**PD-1**	BV650	EH12.2H7	Biolegend
**CD31**	PE	WM59	BD Biosciences
**IFNγ**	BUV395	B27	BD Biosciences
**GM-CSF**	PE	BVD2-21C11	BD Biosciences
**IL-17**	BV421	N49-653	BD Biosciences
**IL-22**	PerCP-eFluor710	22URTI	eBioscience

Antibody	Fluorochrome conjugation	Clone	Source
**IL-10**	APC	JES3-19F1	BD Biosciences
**IL-4**	PE-CF594	MP4-25D2	BD Biosciences
**IL-6**	BV421	MQ2-13A5	BD Biosciences
**TNFα**	BUV395	Mab11	BD Biosciences
**MCAM**	APC	541-10B2	Miltenyi Biotech
**CD49d**	BV421	9F10	Biolegend
**CD11a**	APC-R700	HI111	BD Biosciences
**CD162**	PE	KPL-1	Biolegend
**CD6**	FITC	M-T605	BD Biosciences
**2B4**	PE/Dazzle594	C1.7	Biolegend
**Tbet**	AlexaFluor647	O4-46	BD Biosciences
**EOMES**	Pe-Cy7	WD1928	ebioscience
**Ki-67**	BV421	Ki-67	Biolegend
**CD14**	FITC	M5E2	BD Biosciences
**CD14**	BV786	M5E2	BD Biosciences
**CD56**	PE-CF594	B159	BD Biosciences
**CD16**	APC-Cy7	3G8	BD Biosciences
**CD11c**	PE	B-ly6	BD Biosciences
**CD123**	BV421	9F5	BD Biosciences
**CD86**	APC	2331	BD Biosciences
**LIVE/DEAD Fixable Aqua Dead Cell Stain**	N/A	N/A	Invitrogen
